# Effect of Streptokinase on Reperfusion After Acute Myocardial Infarction and Its Complications: An Ex-Post Facto Study

**DOI:** 10.5539/gjhs.v7n4p184

**Published:** 2014-12-31

**Authors:** Leila Taheri, Ali Zargham-Boroujeni, Marzieh Kargar Jahromi, Maryam Charkhandaz, Mohsen Hojat

**Affiliations:** 1 Faculty of Nursing and Paramedical, Jahrom University of Medical Sciences, Jahrom, Iran; 2 Nursing and Midwifery Care Research Center, Faculty of Nursing and Midwifery, Isfahan University of Medical Sciences, Isfahan, Iran; 3 Jahrom University of Medical Sciences, Jahrom, Iran

**Keywords:** acute myocardial infarction, streptokinase, complications, reperfusio

## Abstract

**Introduction:**

Emergency treatment of patients with acute myocardial infarction is very important. Streptokinase in Iran is often as the only clot-busting medication is used. The purpose of using streptokinase medication is to revive the ischemic heart tissue, although has dangerous complications too. Therefore, the present study aimed to determine the effect of streptokinase on reperfusion after acute myocardial infarction and its complications, has been designed and conducted.

**Materials and Methods:**

This is an Ex-post facto study. The study population included patients who suffer from acute myocardial infarction. The sample size was 300 patients, and 2 groups were matched, in variables of age, sex, underlying disease, frequencies and area of MI.

Data collection did by researcher making questionnaire, that accept face and content validity by 10 expert researcher, the reliability was conducted with Spearman’s test (r=0.85) by Test-retest method. Data analysis did by SPSS software: V 12.

**Findings:**

Mean of EF in SK group was (46.15±8.11) and in control group was (43.11±12.57). Significant relationship was seen between SK, arrhythmia occurring and improve EF reperfusion by chi-square test (p=0.028), (p=0.020). The most arrhythmia in SK group was Ventricular Tachycardia (20.7%). Significant statistical relation between SK and mortality were found by Chi-square test (p=0.001). But a meaningful statistical relation was not found between SK and pulmonary edema incidence (p=0.071).

**Conclusions:**

Nurses of CCU should be aware about SK complications such as hypotension, bleeding and arrhythmias. Proposed compare SK and tissue plasminogen drug in reperfusion and complications effect.

## 1. Introduction

Myocardial infarction in most countries is introduced as the main cause of mortality. In all sources of myocardial infarction, it’s classified among emergency disease (Roger et al., 2012). In the world, a lot of researches have been done on pathological process, causes of incidence and prevalence, risk factors, biological rhythm, treatment, care and rehabilitation of this disease, which has led to the invention of caring methods and special drugs (Perloff & Marelli, 2012). One of these methods is the use of clot-busting drugs such as streptokinase and tissue plasminogen. Among them, streptokinase due to several-years recording of using in Iran is better known and used more. This medicine makes possible increasing of patient survival rate through clot resolution and reperfusion to injured cardiac tissue. However, many side effects such as nausea, vomiting, hypotension, hypertension, phlebitis, localized tenderness, bleeding, bradycardia, tachycardia, arrhythmia and fever also have been reported (Gemmill et al., 1991; Buyukkaya et al., 2012). Considering the high use of this medicine in intensive and emergency wards by nurses, being aware ofthe prevalence and incidenceof the most important complications for promotion of medical team readiness in preventing and treatment of the secomplications on time, is a crucial point in improving the qualityof patient carewho hasacute myocardial infarction (Byrne, 2012; Khan & Mills, 2012). Therefore, the present study aimed to determine the effect of streptokinase on reperfusion after acute myocardial infarction and analyzing its complications have been designed and conducted.

## 2. Materials and Methods

This research is an Ex-post facto study. The research populations were all patients admitted in CCU with a diagnosis of acute myocardial infarction (AMI) with T segment elevation (STEMI) in Motahary Hospital of Jahrom university medical sciences, 2012-2013. Sample size was 150 patients (who received SK) and 150 patients who received conventional treatment of patients with AMI. Those were selected by purposive sampling method. Both groups were matched for age, sex, underlying disease, frequency and area of MI. In all of them cardiologist recognized (STEMI) by using of Creatine Kinase enzyme, Troponin markers and ECG. All patients can include in this study. Exclude criteria were previous arrhythmias, heart failure, liver failure, renal failure and not referring at the first 6 hours after MI. All patients received SK 1500000/U (Farmsya-Belgium) in 1 hour. According to Bruner (2012) patients that have major surgery, receiving SK in earlier 6 months, active bleeding, disorders bleeding, history of hemorrhagic stroke, uncontrolled hypertension and major trauma can’t receive SK. The most difficult step was fined and match control group. Cardiac reperfusion was done by 2 non-invasively determined methods: 1- arrhythmia incidence at the first six hours after acute myocardial infarction and 2- echocardiogram that were reviewed by cardiologist (Rostamzadeh, Khadem, Yekta, & Mohammadzadeh, 2006. Srichaiveth et al., 2007; Masood, Thawerani, & Faruqui, 2012).

## 3. Results

Total number of sample was 300 patients, of which it was 115 (38.33%) female and 185 (61.67%) male the average (SD) age was 56.63 (11.04). 199 (66.33%) patients were lived in city and 101 patients (33.76%) livedinrural areas. 47 patients (67.15%) expiredinthe first daysafteracute myocardial infarction, 12 (8%) patients were of thestreptokinasegroupand 35 (23.3%) inthecontrol group. 40 patients (13.33%) were involved with acute pulmonary edema, that 18 (12%) patients were of the strep to kinase group and 22 (14.66%) in the control group. The mean ejection fraction of patients instreptokinasegroup was 46.15 (8.11) andin the control group was 43/11 (12/57). 147 (49%) patients didn’t have previously diagnosed disease (Table 1). The most common region of MI was in front of the Heart (3.31%) (Table 2), and in 272 (90.7), it was their first myocardial infarction.

**Table 1:** Previously diagnosed disease in patient with acute myocardial infarction

Total		With streptokinase		Without streptokinase		disease

Relative frequency	frequency	Relative frequency	frequency	Relative frequency	frequency	
8.7	26	4.35	13	4.35	13	diabetes
11.3	34	5.3	16	6	18	Heart ischemia
14.7	44	6.3	19	8.4	25	hypertension
1	3	.34	1	.66	2	hyperlipidemia
4.7	14	2.35	7	2.35	7	Diabetes+ ischemia
10.67	32	5.67	18	5	14	Diabetes+ischemia+hypertension
49	147	25.33	76	23.67	71	No disease

**Table 2:** Infarction region of heart in patient admitted to CCU

Total		With streptokinase		Without streptokinase		MI region

Relative frequency	frequency	Relative frequency	Frequency	Relative frequency	frequency	
31.3	94	16.3	49	15	45	frontal
2.7	8	1.35	4	1.35	4	posterior
17.7	53	9	27	8.7	26	inferior
15	45	7	21	8	24	Fronto-lateral
6.3	19	3.3	10	3	9	iferio-posterior
3.7	11	2	6	1.7	5	All regions
11.3	34	5.3	16	6	18	Broad region
6	18	2.7	8	3.3	10	Right inferior
6	18	3	9	3	9	Frontal-septal

The most arrhythmia was in SK Ventricular tachycardia (20.7%). Just in 8 cases (5.3%), it changed to ventricular fibrillation, in control group the ventricular fibrillation was observed in 13 patients (8.7%), it was found out that there is significant statistical relation between SK and decreasing arrhythmia incidence by using Chi-square test (p=0.028) (Table 3). The complications such as arrhythmia, nausea, vomiting, hypotension, bradycardia, and bleeding were noticeably more than the control group and the most common place of hemorrhage; skin, gastrointestinal and urinary systems were reported (Table 4). Two significant statistical relation between SK and mortality were found by Chi-square test (p=0.001). But a meaningful statistical relation was not found between SK and pulmonary edema incidence (p=0.071).

**Table 3:** Types of arrhythmias after Acute Myocardial Infarction

With streptokinase		Without streptokinase		Type of arrhythmia

Relative frequency	frequency	Relative frequency	frequency	
5.3	8	8.7	13	Ventricle fibrillation
20.7	31	6.7	10	Ventricle tachycardia
2.7	4	2.7	4	Atrial fibrillation
2	3	1.3	2	Ventricle-atrial block type 1
2	3	.7	1	Ventricle-atrial block type 2 (venkebakh)
7	1	.7	1	Ventricle-atrial block type2-2
2.7	4	3.3	5	Ventricle-atrial block type 3
5.3	8	2	3	Atrial tachycardia
10	15	6.7	10	Atrial bradycardia
6.7	10	2	3	Intraventriclerdysrhythmia
14.7	22	5.3	8	PVC
27.3	41	60	90	Without arrhythmia

**Table 4:** Complication occurred 6 hours after AMI

Without streptokinase		With streptokinase		complication

Relative frequency	frequency	Relative frequency	frequency	
40	60	76.67	109	arrhythmia
0	0	.7	1	allergy
0	0	6.7	10	bleeding
6	9	21.3	32	vomiting
8	12	21.3	32	nausea
3.3	5	8	12	fever
0	0	0	0	phlebitis
10.7	16	21.3	32	hypotension
13.3	20	10.7	17	bradycardia

## 4. Discussion

Decreasing of mortality rate of patients in SK group than control group is a point that is consistent with Rostamzadeh’sstudy, Sanguanwong and Grajek (Rostamzadeh, Khadem, Yekta, & Mohammadzadeh, 2006; Sanguanwong, Srimahachota, Tungsubutra, SrichaivethB, & Kiatchoosakun, 2007; Grajek et al., 2008). Improving of ejection fraction rate in SK group rather than control group was an event shown in many researches such as Khan’s research (Khan & Mills, 2012).

Regarding to arrhythmia incidence in patients who received SK was more than the control group. But these arrhythmias (just 5.3%) changed to dangerous arrhythmia (ventricular fibrillation) and they needed urgent treatment. This case is consistent with Masood et al. Study that showed the worst arrhythmia after SK injection is ventricular fibrillation (Masood, Thawerani, & Faruqui, 2012). But in control group, although the occurrence of arrhythmia was less and the ventricular fibrillation that needed urgent treatment occurred in 8.7% of cases. The result of recent study is not consistent with Solomon (1993) and Kuijt’s research (2012) in which the occurrence of arrhythmia in patients who used SK was reported less or equal to control group (Solomon, Ridker, & Antman, 1993; Kuijt, Williams, Granger, Krucoff, & Roe, 2012). But particularly about decreasing of the atrium-ventricular blocks variety in those patients who used SK approved the result of Khan’s Study (Khan & Mills, 2012).

The present study as well as Karabag’s study introduces atrium blocks (13%) and atrium-ventricular fibrillation (14%) as one of the created arrhythmia among those patients who used SK and they were removedwithout special treatment (Karabag et al., 2012). Gressin’s treatment study indicated that the most common arrhythmia among AMI patients after using SK is sinus bradycardia (25%), while in the present study the most observed arrhythmia after SK using was tachycardia (20.7%). This result is consistent with Chittari’s study in UK (2005) in which says 21% for incidence of this type of arrhythmia (Chittari et al., 2005; Gressin, Gorgels, Louvard, Lardoux, & Bigelow, 1993). According to the rules and orders of physician, Clemastine antihistamine was prescribed for all individuals before SK injection, it was impossible not to inject the antihistamine because of ethic of research, so the incidence of sensitivity cannot be studied.

The other common side effects in order are: vomiting, hypotension, nausea, hemorrhage, fever, and bradycardia. Sources such as internal medicine of Harrison have announced the least systolic hypotension because of this medicine. This case has been confirmed in Tatu’sstudy (Malani, 2012; Tatu-Chițoiu et al., 2007). It’s important to mention that high systolic blood pressure rate of AMI patients can decrease the mortality of such patient and increase their survival rate. Therefore, low blood pressure as one of the crucial complications of AMI patients’ treatment at the time of using SK should be considered by special wards and emergency nurses (Fauci, 2008).

It’s necessary to mention that the model of hemorrhage in this study were urinary and hidden digestive hemorrhage and also skin hematoma that is not consistent with both model and incidence rate of hemorrhage in Tatu’s study (2007) in which the model of hemorrhage has been expressed that it is intracranial hemorrhage (25%) by considering the incidence order. This difference can be due to demographic features of different patients (Tatu-Chițoiu et al., 2007).

Finally, it can be said that the incidence rate of injection complications in SK group is more than the control group.

## 5. Conclusion

Nurses of CCU should be aware about SK complications such as hypotension, bradycardia, fever, nausea, vomiting, bleeding and arrhythmias. But theincreased incidence ofarrhythmiasand improvedejection fractionin thestreptokinasegroupcompared tothe control group (promotion of twonon-invasivemarkersof reperfusion of the injured hearttissue), alsoreduced the risk of dangerous arrhythmias and reduced mortality in patients with acute myocardial infarction can indicate the importance of using this drug.

At the end, it is proposed to compare the reperfusion effect and complications of streptokinase versus the tissue plasminogen drug inpatients with acute myocardial infarction in terms of a comparative study.
